# Advances in Understanding the Pathogenesis of Craniofacial Birth Defects

**DOI:** 10.3390/jdb10030027

**Published:** 2022-07-01

**Authors:** Andre L. P. Tavares, Sally A. Moody

**Affiliations:** Department of Anatomy & Cell Biology, George Washington University School of Medicine and Health Sciences, Washington, DC 20052, USA; tavaresa@gwu.edu

Each year approximately 35% of babies are born with craniofacial abnormalities of the skull, jaws, ears, and/or teeth, which in turn can lead to problems in feeding, hearing, and sight. Because of this high incidence and the clinical importance of correcting skeletal dysmorphologies by surgical interventions, the developmental biology and pediatric research communities have been highly engaged in investigating how these tissues form during normal development and discovering the genetic underpinnings of the hundreds of distinct congenital craniofacial syndromes. A large body of previous research has elucidated many of the cellular and molecular processes that underlie the normal interactions between the various embryonic cell types that contribute to these tissues as well as the genes and signals that are aberrant in craniofacial syndromes. In recent years, however, research has focused on using advanced genomic and transcriptomic approaches to screen patients for causative variants, high resolution imaging, and morphometrics to describe phenotypes more accurately, and animal models that harbor human syndromic variants. Many of these advances are exemplified in the studies that comprise this Special Issue on “Craniofacial Genetics and Developmental Biology”, revealing many new insights into normal and aberrant craniofacial development.

One of the major embryonic contributors to the craniofacial tissues is the cranial neural crest, a pluripotent population of cells originating from the border of the neural plate that migrate away from the neural tube as it closes and migrate into the periphery to form multiple craniofacial tissues, including the anterior skull and the skeleton of the face. In this Special Issue, Siismets and Hatch [1] review our current understanding of the contribution of neural crest to craniofacial development and various craniofacial anomalies, and then focus on the pathogenesis of coronal craniosynostosis, the premature closure of the coronal suture that causes significant clinical outcomes. The authors also discuss potential approaches for craniofacial tissue regeneration as well as treatment for craniosynostosis. Weigele and Bohnsack’s review [2] focuses on the key role that the cranial neural crest plays during ocular development and congenital eye diseases. Neural crest interactions with the periocular mesenchyme and the neural tube-derived optic cup are critical for ocular morphogenesis; defects in these interactions result in microphthalmia and coloboma. Cranial neural crest cells also contribute to the cornea, iris, sclera, ciliary body, trabecular meshwork, and aqueous outflow tracts; defects in their migration and differentiation can cause numerous anomalies in these ocular structures, such as Axenfeld-Reiger Syndroma and Peters Anomaly. Another study in this Special Issue used the forward-genetics approach of ENU mutagenesis in mouse to identify a new player in ocular development [3]; Blizzard et al. discovered a new, hypomorphic allele of *Cse1l*, whose wildtype protein functions in several cellular processes including nuclear transport, cell cycle, and apoptosis. The previously reported *Cse1l*-null mutant is early embryonic lethal, whereas the hypomorphic *Cse1l* mutants described in this report survive to organogenesis stages, presenting with a number of variable craniofacial and ocular phenotypes including microphthalmia and coloboma. While embryonic *Cse1l* expression is widespread, it is highly expressed in the migrating cranial neural crest, consistent with the affected tissues. The authors suggest that this new allele provides evidence that CSE1L should be considered in patients with eye and craniofacial abnormalities of unknown cause.

Defects in components of the cranial skeleton are a hallmark of a number of craniofacial syndromes. Many syndromes include dysmorphologies of the cranial vault, called the calvarium. The anterior portion of the calvarium is derived from the cranial neural crest, whereas the posterior portion derives from paraxial mesoderm. In this Special Issue, Parmar et al. [4] studied the role of cyclooxygenase-2 (Cox2) in calvarial development; Cox2 catalyzes the formation of prostaglandin E2 (PGE2) which is required for cell proliferation, migration, epithelial-mesenchymal transition, and differentiation in a variety of tissues. They found that loss of Cox2 leads to abnormal calvarial development. Reducing Cox2 activity reduced the level of PGE2, which in turn led to reduced expression of cell adhesion molecules (E- and N-cadherins) and their regulators (Msx1, Tgf-beta) that are necessary for normal migration of the neural crest into the calvarial primordium. In another study of calvarial development, Ibarra et al. [5] experimentally demonstrated that coordination between Wnt/beta-Catenin and Erk signaling regulates the adoption of cartilage versus bone fate in the neural crest-derived cranial mesenchyme that forms the calvarium. Loss of Erk signaling led to ectopic cartilage in the frontal bone primordium indicating a shift from osteogenic fate to chondrogenic fate. These two studies significantly contribute to our understanding of the molecular control of calvarial development.

Another prominent class of dysmorphologies are those that affect the midface, jaws, and teeth. Liberton et al. [6] tested whether dentofacial deformities known as skeletal malocclusions contribute to the craniofacial morphology of non-syndromic cleft lip and palate patients. By comparing the geometric morphometry data obtained by advanced imaging—full skull cone beam computed tomography—of sex- and ethnicity-matched patients, these authors found that both the cleft and the malocclusion contribute to the extent of the craniofacial phenotype. Dasgupta et al. [7] report that R-spondins, secreted proteins that augment Wnt signaling, are required for the formation of lower jaw incisors. Using compound mutant mice, they showed that simultaneous deletion of two members of the R-spondin family led to hypoplasia of the mandible and cleft secondary palate as well as severe dental abnormalities. This paper is the first evidence that R-spondin signaling promotes the normal process of odontogenesis in mammals. Ko et al. [8] investigated the mechanism by which teeth and jaws fit, function, and evolve together. They tested whether molar teeth only begin to grow once the jaw develops enough space from the previously erupted tooth. Using another advanced imaging technique—synchrotron-based micro-CT scanning—they assessed developing molars in the mouse jaw from E10 to P32. They found that conditions within the dental lamina itself, rather than jaw growth, have the greater influence on molar spacing. Their data support the conclusion that molar initiation is contingent on sufficient surface area for the dental epithelium to reorganize and invaginate into the underlying mesenchyme.

The cranial base, which is derived from both neural crest (anterior elements) and paraxial mesoderm (posterior elements), has been less studied than the calvarium and jaws. However, recent work shows that defects in its development can have widespread craniofacial consequences. In the review by Venugopalan and Van Otterloo [9], the authors focus on the signals and genes that regulate the development of the cranial base in animal models and humans. Unlike the skeletal elements of the calvarium and face, which develop by intramembranous ossification, the cranial base skeleton develops from an intermediate chondrocranium similar to the long bones of the trunk. These authors compare the gene regulatory networks that may differ between these two modes of bone formation and pose many questions regarding their roles in evolutionary diversity of shape and congenital dysmorphologies. Because craniofacial birth defects often include anomalies of the cranial base, it is important to elucidate the developmental regulation of this region of the skeleton. In support of this conclusion, Kidwai et al. [10] present a case report of Muenke syndrome (MS), a disease caused by the p.Pro250Arg variant in FGFR3 and characterized by coronal suture synostosis, macrocephaly, dysmorphic craniofacial features, and dental malocclusion. However, because phenotypes are highly variable and penetrance is incomplete, it is difficult to unravel the cellular and molecular mechanisms underlying MS. To establish a rigorous phenotypic framework to account for skeletal phenotypic variance, the authors quantitatively delineated the craniofacial phenotype of an individual with MS and compared this to his unaffected parents using 3-dimensional cephalometric analysis of cone beam computed tomography scans and geometric morphometric analysis. The measurements of the proband illustrated a shortened anterior and middle cranial base. Interestingly, while measurements of both unaffected parents were within the normal clinical range, they were at variance with a separate, healthy control dataset, suggesting while they do not carry the mutation, they may carry modifiers that contribute to the phenotype. These results suggest that clinically unremarkable, but shortened cranial base bones likely have downstream effects on the other craniofacial phenotypes. This study highlights how deep morphological assessment in both affected and unaffected family members can lead to more focused research questions and examine the impact of genetic variants on craniofacial development.

This Special Issue also presents work that addresses the function of variants found by GWAS and next-generation sequencing to be causative in a few human craniofacial syndromes. Motch Perrine et al. [11] review the current understanding of the highly variable phenotypes associated with Pierre Robin syndrome. Basing their study on these phenotypes, which include small jaws, tongue displacement, and cleft palate, and on our knowledge of how the affected tissues develop, the authors provide a list of genes whose misregulation may contribute to Pierre Robin syndrome. They also provide a list of the available animal models that could be used to better understand the genetic basis and phenotype variation in this syndrome. In the MS case report cited above [10], the authors generated iPSCs from each member of the family trio to try to understand how the p.Pro250Arg variant of the proband affects the function of FGFR3. The structural changes in the MS receptor are predicted to confer greater promiscuity for atypical ligands. Using a novel imaging approach—two-photon fluorescence lifetime imaging microscopy—the autofluorescence decay of specific amino acids data collected from the iPSC cells supported this prediction by showing a shorter half-life for the MS FGFR3 compared to the wild-type FGFR3 from either parent. The authors posit that these cell lines will serve as a reliable, patient-specific platform with which to understand the cellular processes that are affected in MS. The variants associated with another craniofacial syndrome, Branchio-Oto-Renal (BOR) syndrome, were investigated by Mehdizadeh et al. [12]. They expressed the homologues of four different human variants of *SIX1* (BOR) in wild-type *Xenopus* embryos to model this autosomal dominant disease in which patients express one wild-type allele and one mutant allele. Previous work from this laboratory demonstrated that variants of either the cofactor-binding domain or the DNA-binding domain each altered—in unique combinations—gene expression in both the neural crest and sensory placodes at neural plate stages. In this study, they focused on how these variants affect gene expression in the primordium of the inner ear—the otic vesicle. The four different single nucleotide variants, each of which has defective transcriptional activity, showed different effects on a suite of otic genes. The authors propose that these differences arise from differences in the ability of cofactors to bind to each variant, which then differentially interfere with their ability to drive otic gene expression, which ultimately may contribute to patient phenotype variability.

This exciting collection of articles that address fundamental issues in normal and aberrant craniofacial development demonstrates the unexpected new information that can be obtained by using new methodologies and several different appropriate animal models. Relating the fundamental information to the clinical literature and case reports promises to advance treatment for these all-too-frequent syndromes.
